# Is There Truly No Benefit with Sunscreen Use and Basal Cell Carcinoma? A Critical Review of the Literature and the Application of New Sunscreen Labeling Rules to Real-World Sunscreen Practices

**DOI:** 10.1155/2012/480985

**Published:** 2012-05-09

**Authors:** Cameron Chesnut, Jenny Kim

**Affiliations:** Division of Dermatology, David Geffen School of Medicine at UCLA, V A Greater Los Angeles Healthcare System, 52-121 CHS, Los Angeles, CA 90095, USA

## Abstract

Basal cell carcinoma (BCC) is the most common human malignancy. Both epidemiological and direct evidence have established ultraviolet (UV) exposure from the sun as the most important risk factor for BCC development. There has only been one randomized and controlled study to examine sunscreen's role in the prevention of BCC, and no significant protective benefit was found. This study did not address four important concepts: sunscreen abuse, sunscreen misuse, sunscreen formulation, and cumulative UV exposure. Thus, the results of this study are difficult to interpret and extrapolate with real-world sunscreen practices.

## 1. Introduction

Basal cell carcinoma (BCC) is the most common cutaneous malignancy, the most common human malignancy overall, and its incidence is increasing [1]. For more than 80 years, convincing epidemiological evidence has linked sun exposure with skin cancer. BCC is more frequent in patients with higher cumulative sun exposure, with more sun-sensitive skin types, from areas of high ambient solar irradiance, and on sun-exposed body sites [1, 2].

Both the quality and quantity of such epidemiological evidence have improved over those 80 years, and more recent data have given us direct evidence of ultraviolet- (UV-) induced mutations in genes important to BCC development. UV-induced mutations in the p53 tumor-suppressor gene have been found in more than half of BCC cases [3]. Mutations that activate the Hedgehog intercellular signaling pathway genes, including PTCH, Sonic hedgehog, and Smoothened, play a significant role in the development of BCC, and these mutations have also been shown to be UV induced [4].

UVB exposure seems to be the most important risk factor in developing BCC [2, 4–7]. Rats exposed to UVB develop BCC as well [8]. UVA has also been implicated as a risk factor for BCC, including exposure during the use of tanning beds [9, 10].

## 2. Evidence of Sunscreen and BCC

With the propensity of both epidemiologic and direct evidence indicating the chief role of UV in the development of BCC, one may expect a similar propensity of evidence indicating that UV blockade by sunscreen protects against BCC. To date, there has only been one randomized controlled trial examining sunscreen's role in the prevention of skin cancer, and it showed no significant protective benefit of sunscreen with relation to BCC [11].

Green et al.*'*s 1999 study followed 1621 Australian patients for 4.5 years. There was both a daily SPF 16 sunscreen group and a no-sunscreen group that included no placebo lotion. At the end of this trial, 75% of the daily sunscreen group patients were applying sunscreen to their head, neck, arms, and hands 3 or 4 days per week, and this correlated well with the measured weight of sunscreen at scheduled study-clinic visits. In the no-sunscreen group, 74% of patients were not using sunscreen at all or no more than 1-2 days per week [11]. The no-sunscreen group was in fact allowed to wear sunscreen, and 26% of that group was using sunscreen more than 2 days per week.

Skin cancer diagnoses were obtained by scheduled study-clinic visits 2 and 4 years after the initiation of the study, utilizing blinded dermatologists to make clinical diagnoses, which were also confirmed histologically. Diagnoses of skin cancers made by study patients' local doctors at other times were obtained and confirmed with review of medical records.

The use of the SPF 16 sunscreen to the head, neck, arms, and hands had no effect on the incidence of BCC tumors or the total number of BCC tumors occurring at these sites. The incidence of squamous cell carcinoma was significantly reduced in the daily sunscreen group [11]. To assess latency of the protective effects of sunscreen, an 8-year follow-up study of all patients included in the original study was performed by Green and colleagues. Again, a trend, but not a significant reduction in the total number or incidence of BCC, was observed with daily sunscreen use. When isolated to the late follow-up period alone, there was a non-significant reduction in BCC incidence with daily sunscreen users (RR 0.75, 95% CI 0.49–1.14), indicating a possible trend towards decreased BCC incidence over longer time periods [12].

## 3. Discussion: Real-World Sunscreen Practices

Green et al.*'*s data did not support what years of prior epidemiological and direct evidence would have predicted: a protective benefit against BCC by using sunscreen. However, there are four important real-world concepts that were not addressed by this study: sunscreen abuse, sunscreen misuse, sunscreen formulation, and cumulative UV exposure.

The idea of sunscreen abuse has been elucidated by three randomized trials in Europe that examined the behavior of subjects on vacation using sunscreen with known SPF values. These studies elucidated the idea of intentional sun exposure (ISE) and nonintentional sun exposure (NISE). ISE is sun exposure with an intention to stay in the sun with large areas of uncovered skin or to acquire a tan [13]. These three trials found that the subjects with ISE had differing patterns of behavior with different SPF sunscreens assigned for use. Higher known SPF values were associated with a dramatic increase in time spent in the sun, specifically sunbathing [14–16]. Higher SPF values were also associated with dramatic decreases in the amount of sunscreen used, the amount of clothing used to cover up while in the sun, and the time of day when sun exposure was obtained, with the high SPF group sunbathing closer to the noon hours. The abuse of sunscreen occurs because sunburn is delayed, and the users are able to practice sun exposure behavior that would not be possible if they were burning. The users' perceived endpoint, sunburn, is thus delayed but still obtained [13].

In Green's trial, the daily sunscreen group was aware that they were using sunscreen, and the nonsunscreen group had no placebo, and thus they knew that they were not being protected. According to the three aforementioned studies, this knowledge alone would have significantly altered the sun exposure behavior practices of the two groups.

Sunscreen misuse is nearly ubiquitous when respect is given to the fact that 2 mg/cm^2^ is the amount of sunscreen used when SPF is measured. Studies examining the actual amount applied by most users have revealed amounts closer to 0.5 mg/cm^2^, or one quarter of the recommended amount, which yields an effective SPF of about 1/3 the labeled SPF [17]. Theoretical calculations suggest that there is an exponential relationship between SPF and the amount of sunscreen used, and that exponential growth has been suggested by studies in human skin [17]. Green's study did not mention the actual amounts of sunscreen applied, but with a SPF 16 sunscreen and the realistic amounts used by the subjects, the actual SPF obtained was likely exponentially lower than 16. The other form of blatant sunscreen misuse in Green's study was the fact that in the daily sunscreen group, 75% of patients were applying sunscreen to parts of their body 3-4 days per week, and this was supported by measured sunscreen weights [12]. In the “daily” group, 75% of the subjects were using sunscreen roughly 50% of the time, and one may consider that more consistent use of the daily assignment may have yielded different results. In addition, the control group was allowed to wear sunscreen, and 26% of that no-sunscreen group was in fact wearing sunscreen more than 2 times per week. Thus, the actual protective benefit is likely far greater than that stated in Green's study.

In the 1990s, suspicion of UVA's role in cutaneous carcinogenesis fueled an explosion of new “broad spectrum” sunscreens [13]. One may be hard pressed to find a sunscreen resembling the antiquated formulation used in Green's study on a shelf for sale today. Green's study formulation did utilize avobenzone but had no concurrent UVA stabilizer [11]. With today's standards, both the low SPF and lack of stable UVA coverage provided by this sunscreen may cause providers and consumers alike to shy away. With the new US Food and Drug Administration (FDA) labeling standards, this sunscreen from Green's study would have to live up to its purported SPF exactly to claim that it prevents skin cancer; if it is tested under SPF 15, it could only claim to prevent sunburn [18]. With issues over the stability of its UVA coverage, it may not meet the standards that today's sunscreen formulations are required to reach to be labeled broad spectrum [18, 19]. Unfortunately, no randomized trials exist to date examining what effect a newer sunscreen formulation may have with relation to basal cell carcinoma and other skin cancer development.

The likelihood of developing BCC increases with age, having a median age at diagnosis of 67 years and a mean age of 64.4 years [20]. The most common age group affected is 50–80-year-old individuals, with sun damage starting to accumulate at an early age and likely not manifesting as BCC for upwards of 20 years, thus there is a very significant lag time between sun exposure and development of BCC [20]. With such an apparent contribution of cumulative UV exposure, it brings to question how applicable a 4.5-year and even an 8-year extended follow-up period is with respect to actual BCC risk, when patients have likely been accruing UV-induced damage for triple that amount of time. A nonsignificant downward trend was noted in BCC when isolated in the extended 8-year endpoint of Green's study [12], and a longer period of sunscreen use followed over a more substantial time frame may very well have shown a statistically significant benefit.

## 4. Conclusion: Real-World Applications

UV damage causes BCC, and sunscreens block UV. Dermatologists recommend to their patients the daily use of high SPF and broad spectrum sunscreens applied in liberal amounts as part of a photoprotection plan, but no studies have examined the effects of sunscreen on BCC with parameters comparable to these recommendations. Published literature finding no effect of sunscreen on BCC did not utilize true daily use of high SPF and broad spectrum sunscreens with consistent application and likely involved inadequate amounts applied.

The FDA has addressed some of these issues in its new rules for sunscreens. By the summer of 2012, sunscreens will have to provide a minimum SPF of 15 and pass the new broad spectrum test procedure to claim to protect against cancer when used with other methods of photoprotection; if they only achieve one of these two standards, they can only claim to prevent sunburn [18]. Sunscreens will also not be able to claim to provide more than two hours of protection or provide protection immediately after application unless they submit data to prove these claims and are approved by the FDA [18]. All sunscreens will require directions on when to reapply, and sunscreens that are not water resistant will have to instruct consumers to seek water resistant options if swimming or sweating [18].

When viewed as a whole, the FDA's new rules have addressed many of the real-world concepts that have been problematic with sunscreen use in the past. Sunscreen abuse, sunscreen misuse, and cumulative UV exposure have been addressed with mandatory and deliberate directions on the label about reapplying, not providing more than two hours of protection, and sunscreen only being part of a photoprotection plan. The improved capabilities of sunscreen formulations have been respected by requiring both a mandatory SPF minimum and a tested UVA minimum to claim broad spectrum coverage and prevent skin cancer.

While the conclusions of the studies performed to date cannot be extrapolated to real-world physician-patient practices that have proper patient education and compliance, the frontier for research with new sunscreen formulations under new standards and practices is exciting. The field of dermatology can be hopeful that the true role of sunscreen in the prevention of UV-induced skin cancer may soon be reliably understood.
